# Capecitabine reverses tumor escape from anti-VEGF through the eliminating CD11b^high^/Gr1^high^ myeloid cells

**DOI:** 10.18632/oncotarget.24811

**Published:** 2018-04-03

**Authors:** Toshiki Iwai, Yui Harada, Hiroshi Saeki, Eiji Oki, Yoshihiko Maehara, Yoshikazu Yonemitsu

**Affiliations:** ^1^ Department of Surgery and Science, Graduate School of Medical Sciences, Kyushu University, Fukuoka, Japan; ^2^ R&D Laboratory for Innovative Biotherapeutics, Graduate School of Pharmaceutical Sciences, Kyushu University, Fukuoka, Japan; ^3^ Product Research Department, Kamakura Research Laboratories, Chugai Pharmaceutical Co., Ltd., Kanagawa, Japan

**Keywords:** anti-VEGF, capecitabine, myeloid-derived suppressor cells, tumor angiogenesis, pyrimidine nucleotide phosphorylases

## Abstract

The anti-VEGF humanized antibody bevacizumab suppresses various malignancies, but tumors can acquire drug resistance. Preclinical studies suggest myeloid-derived suppressor cells (MDSCs) may be associated with tumor refractoriness to anti-VEGF treatment. Here we report a novel mechanism of tumor escape from anti-VEGF therapy. Anti-VEGF treatment enhanced intratumoral recruitment of CD11b^high^/Gr-1^high^ polymorphonuclear (PMN)-MDSCs in anti-VEGF-resistant Lewis lung carcinoma tumors. This effect was diminished by the anticancer agent capecitabine, a pro-drug converted to 5-fluorouracil, but not by 5-fluorouracil itself. This process was mediated by enhanced intratumoral granulocyte-colony stimulating factor expression, as previously demonstrated. However, neither interleukin-17 nor Bv8, which were previously identified as key contributors to anti-VEGF resistance, was involved in this model. Capecitabine eliminated PyNPase-expressing MDSCs from both tumors and peripheral blood. Capecitabine treatment also reversed inhibition of both antitumor angiogenesis and tumor growth under anti-VEGF antibody treatment, and this effect partially inhibited in tumors implanted in mice deficient in both PyNPases. These results indicate that intratumoral granulocyte-colony stimulating factor expression and CD11b^high^/Gr-1^high^ PMN-MDSC recruitment underlie tumor resistance to anti-VEGF therapy, and suggest PyNPases are potentially useful targets during anti-angiogenic therapy.

## INTRODUCTION

Angiogenesis drives tumor progression [1], and pathways involving vascular endothelial growth factors (VEGFs) and its receptors (VEGFRs) promote tumor angiogenesis [2]. Bevacizumab (Bev) is a humanized anti-VEGF-A neutralizing monoclonal antibody, and the combination regimen of add-on Bev with pre-existing chemotherapeutic agents provide improved clinical benefits more than chemotherapy alone in several malignancy types [3–5]. Unlike chemotherapeutic agents, the anti-angiogenesis strategy should result in far less drug resistance [6, 7]. However, Bev-combined chemotherapy still could not overcome drug resistance in clinical settings [8, 9].

CD11b^+^/Gr-1^+^ cells, which include neutrophils, macrophages, and myeloid-derived suppressor cells (MDSCs), promote tumor escape from anti-VEGF therapy [10, 11]. This process likely involves at least two independent mechanisms: bypassing anti-VEGF-mediated anti-angiogenesis via secretion of the alternative angiogenic factor Bv8 [12] and a MDSC-mediated, Th17-dependent immune suppressive pathway [13]. Both mechanisms were shown to be mediated via intratumoral recruitment of myeloid cells by granulocyte-colony stimulating factor (G-CSF) [12, 13]. In the present study, we used a mouse model to investigate a possible alternative pathway that may promote tumor resistance to anti-VEGF therapy.

## RESULTS

### Anti-VEGF treatment increased the number of intratumoral CD11b^high^/Gr-1^high^ cells

**Figure 1:**
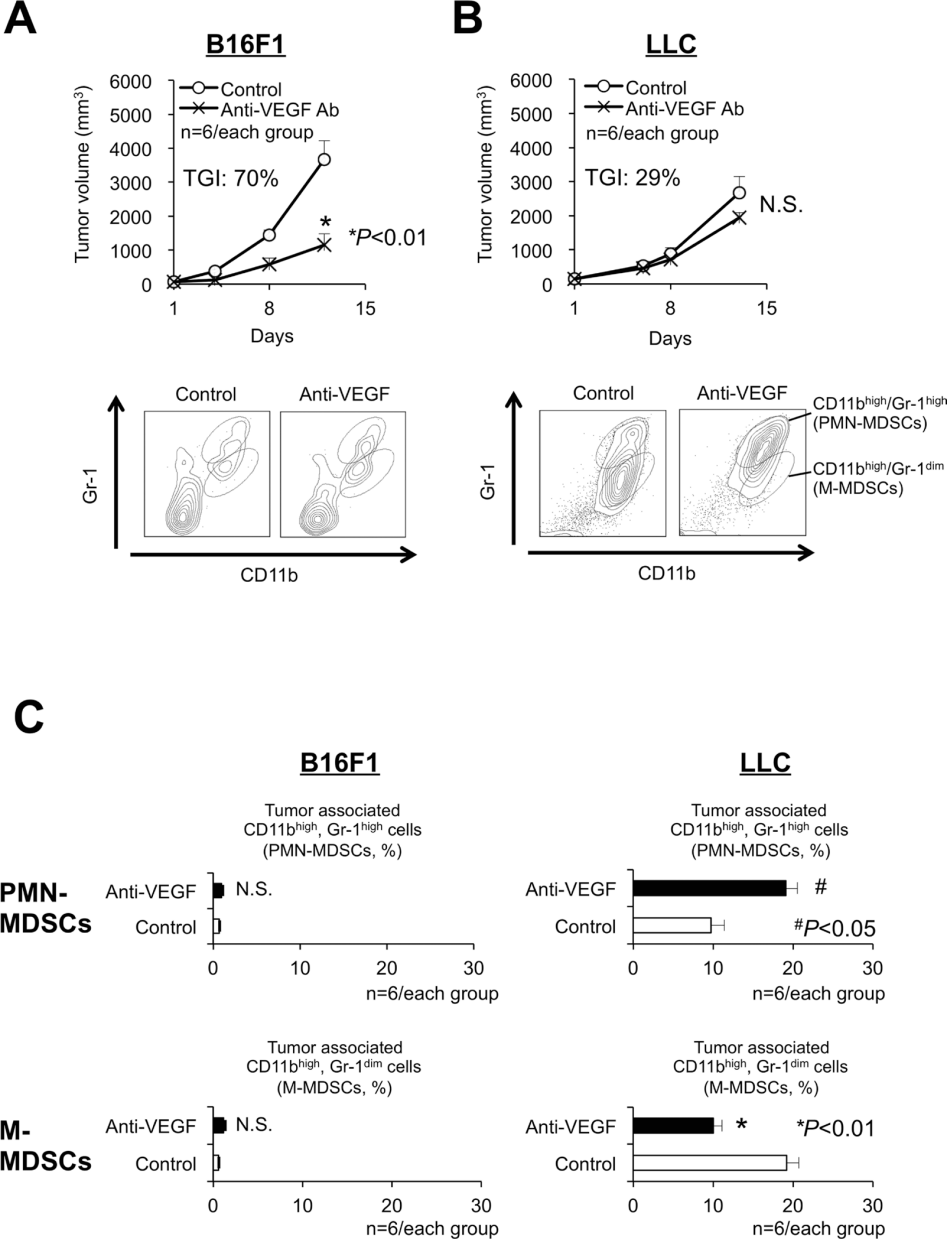
Antitumor responses and accumulation of myeloid-derived suppressor cells (MDSCs) related to anti-VEGF treatment in sensitive or resistant tumors (**A**, **B**) Growth curves (upper graphs, TGI: tumor growth inhibition) and flow-cytometric analyses of the infiltration of MDSCs (bottom graphs) in anti-VEGF-sensitive (B16F1, TGI at day 12 = 70%) (A) and anti-VEGF-resistant (LLC, TGI at day 12 = 29%) (B) tumors in C57BL/6 mice. Note that 2-independent populations (CD11b^high^/Gr-1^high^ or CD11b^high^/Gr-1^dim^) were identified as polymorphonuclear (PMN)- and monocytic (M)- MDSCs, respectively. (*n* = 6/group). (**C**) Quantification of the composition ratio of intratumoral CD11b^high^/Gr-1^high^ cells (PMN-MDSCs) during anti-VEGF treatment. All data are shown as the mean ± SEM. N.S.: not significant, ^*^P < 0.01, and ^#^*P* < 0.05. (*n* = 6/group).

Shojaei *et al.* used B16F1 melanomas and LLC murine lung carcinomas to show resistance to anti-VEGF antibody treatment is associated with intratumoral MDSC accumulation [11]. Because Gr-1 is a cell surface marker that reflects the immune suppressive activity of MDSCs in tumor models [14–16], we used CD11b and Gr-1, rather than Ly6G/Ly6C, to identify the subpopulation of MDSCs. The anti-VEGF treatment was effective against B16F1 tumors associated with a very small amount of CD11b^high^/Gr-1^high^, (Figure 1A) [14–16]. In contrast, the anti-VEGF treatment accelerated intratumoral MDSC accumulation (Figure 1B).

Next, because MDSCs show at least two distinct phenotypes, namely PMN- and monocytic (M)-MDSCs, we investigated the effect of anti-VEGF treatment on these populations using LLC tumors. Anti-VEGF treatment increased the ratio of CD11b^high^/Gr-1^high^ PMN-MDSCs (Figure 1C).

### Capecitabine, a prodrug of 5-FU, restored the antitumor effect of anti-VEGF

**Figure 2:**
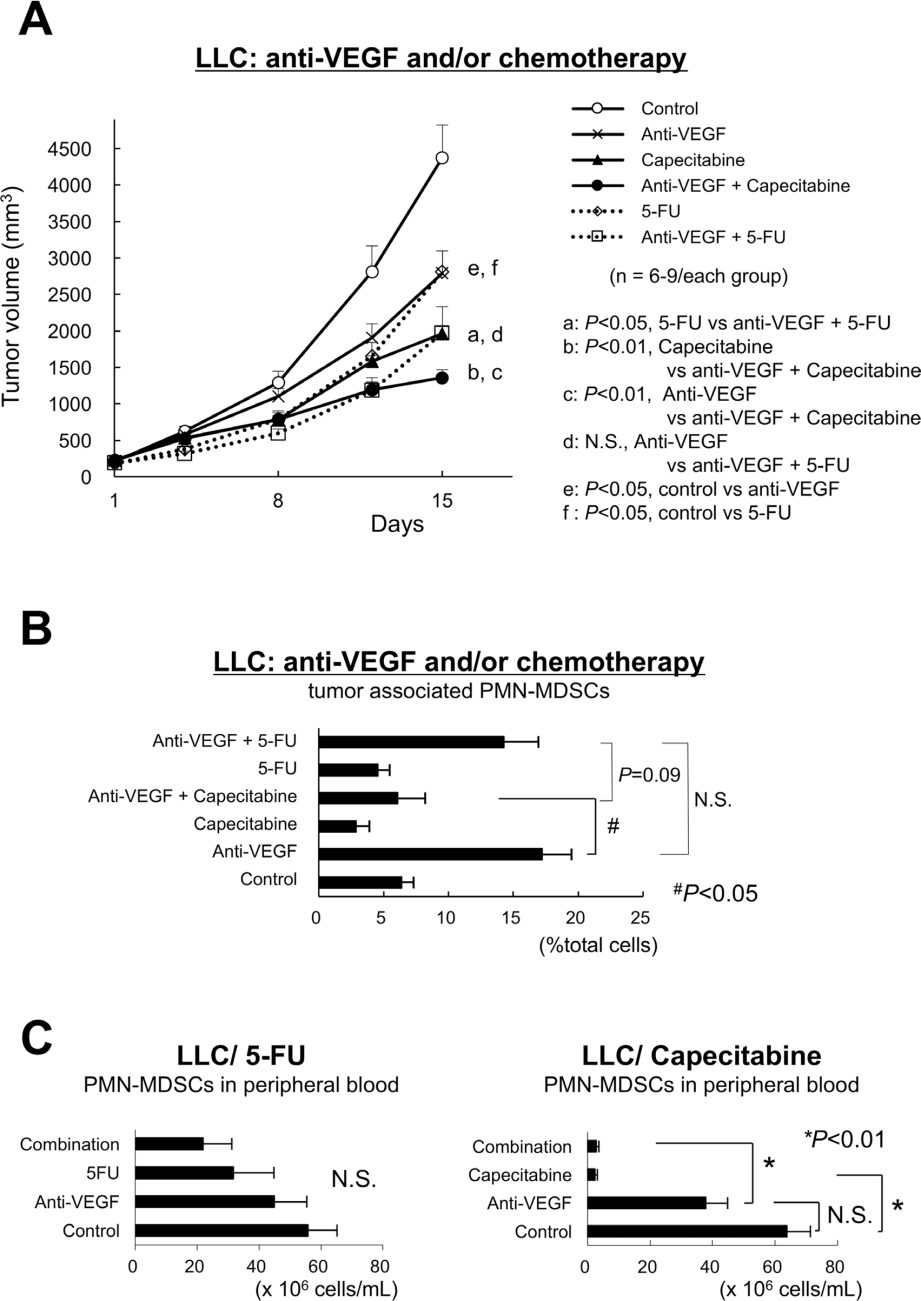
Capecitabine, a prodrug of 5-FU, restores restored the antitumor effect of anti-VEGF (**A**) Sole and combination effects of anti-VEGF, 5-FU and capecitabine on the growth of anti-VEGF -resistant LLC tumors *in vivo*. Note that capecitabine, but not 5-FU, demonstrated a combined antitumor effect with anti-VEGF. (*n* = 6–9/group). (**B**) Sole and combination effects of anti-VEGF, 5-FU and capecitabine on the intratumor accumulation of PMN-MDSCs in anti-VEGF-resistant LLC tumors. Note that treatment with capecitabine, but not 5-FU, resulted in a significant reduction of the intratumor accumulation of PMN-MDSCs. Data are shown as the mean ± SEM (*n* = 6/group). N.S.: not significant, ^#^*P* < 0.05. (*n* = 6/group). (**C**) Effect of 5-FU or capecitabine on the number of circulating PMN-MDSCs. Note that capecitabine, but not 5-FU, almost eliminated PMN-MDSCs in the peripheral blood. Data are mean ± SEM. N.S.: not significant, ^#^*P* < 0.05. (*n* = 6/group).

Since 5-FU can selectively kill MDSCs and enhance T-lymphocyte-mediated antitumor immune responses [17], we evaluated the effect of 5-FU and the clinically available prodrug of 5-FU, capecitabine, on LLC tumor growth under anti-VEGF treatment. Capecitabine, but not 5-FU, demonstrated a combined antitumor effect with anti-VEGF (Figure 2A). In addition, capecitabine diminished the intratumoral accumulation of PMN-MDSCs (Figure 2B) and circulating PMN-MDSCs (Figure 2C, right). 5-FU only partially inhibited these same parameters (Figure 2C, left).

### G-CSF, but neither IL-17 nor Bv8, promoted intratumoral PMN-MDSC recruitment and antitumor angiogenesis in the LLC tumor model

**Figure 3:**
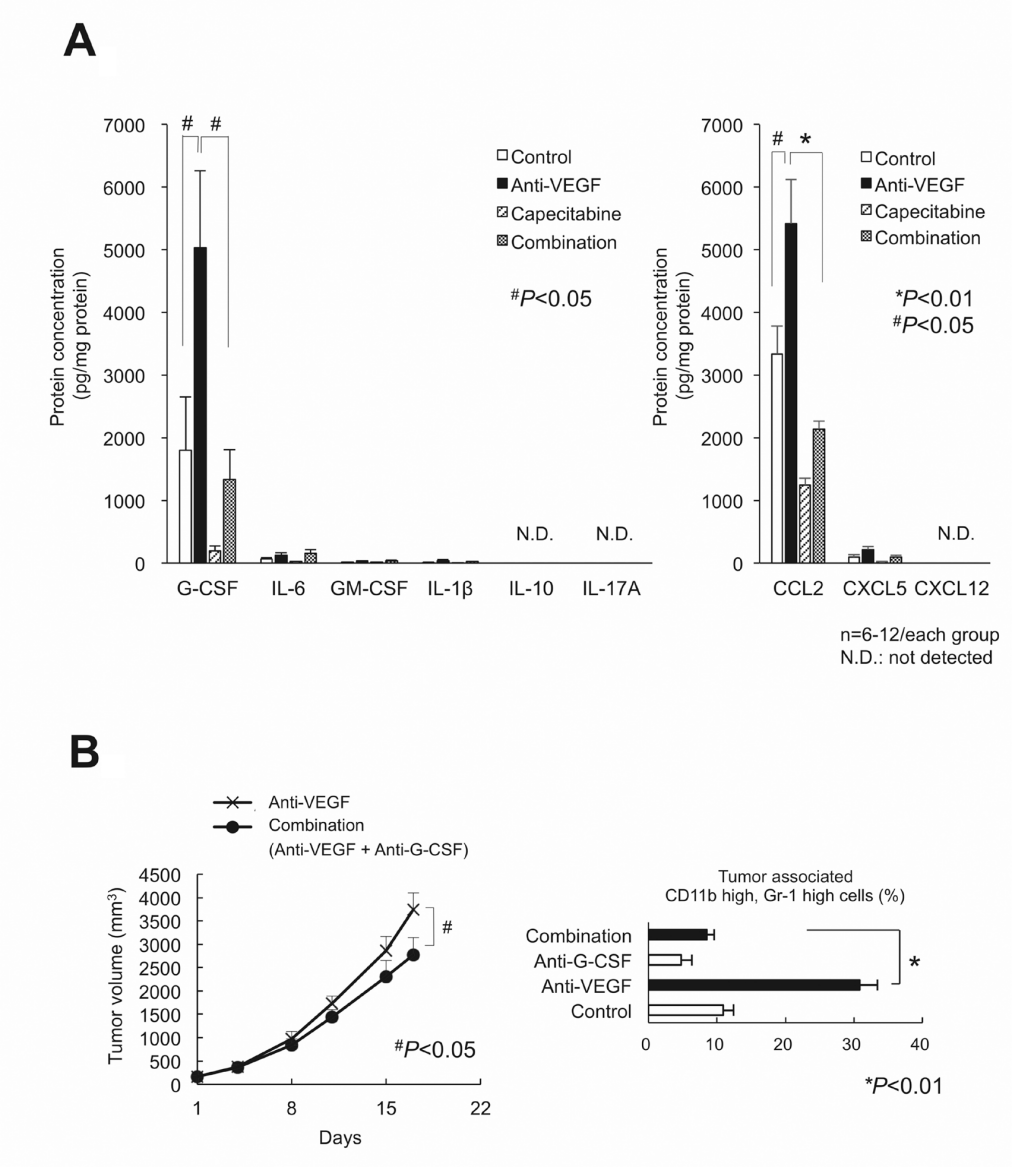
Screening for the cytokines/chemokines in LLC tumor lysate that were affected by anti-VEGF, capecitabine, and their combination treatments (**A**) The screening results by bead array assay (left) or ELISA (right). Only G-CSF and CCL2 exhibited both the increased by anti-VEGF and decreased by capecitabine. IL-17 protein was under the detection limit. (*n* = 6–12/group). (**B**) Effect of neutralization of G-CSF on LLC tumor growth (left) and the intratumor accumulation of PMN-MDSCs (right) during anti-VEGF treatment. The increase of PMN-MDSCs by anti-VEGF was completely cancelled by anti-G-CSF treatment. Data are mean ± SEM. ^*^P < 0.01. (*n* = 6/group)

**Figure 4:**
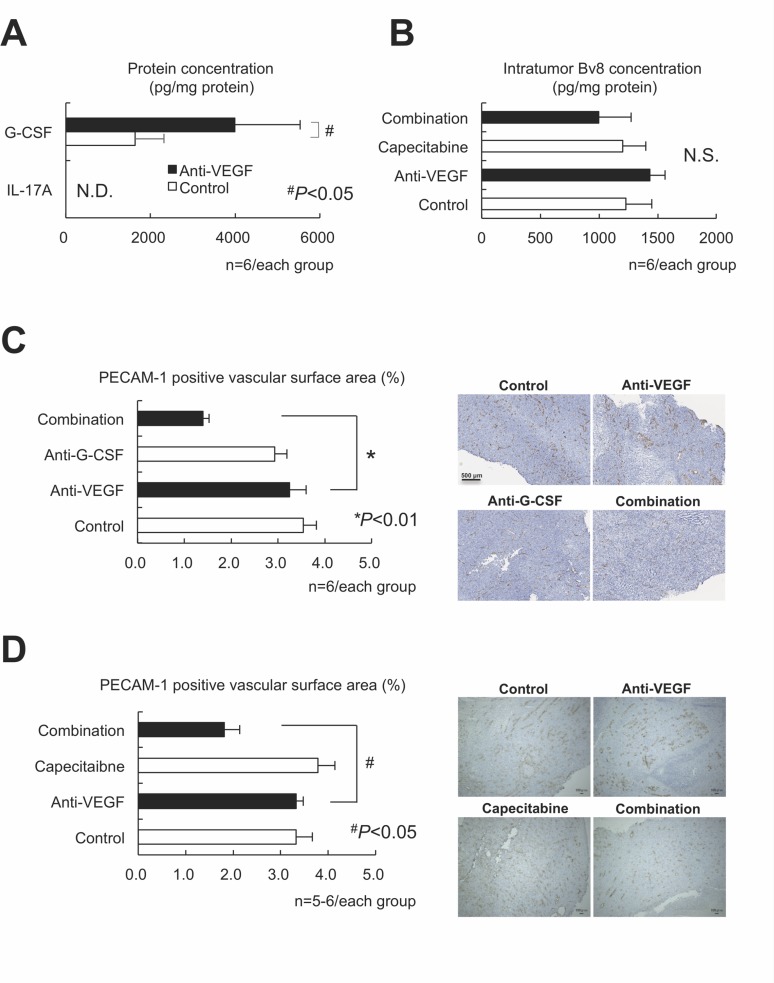
G-CSF, but neither IL-17 nor Bv8, was still essential to the intratumor recruitment of PMN-MDSCs and antitumor angiogenesis in the LLC tumor model (**A**) Confirmation that G-CSF, but not IL-17, was involved in anti-VEGF-mediated upregulation in LLC tumor lysate assessed by ELISA assay. (*n* = 6/group). (**B**) Neither anti-VEGF nor capecitabine affected Bv8 expression as assessed by ELISA. (*n* = 6/group). (**C**) Effects of anti-VEGF, anti-G-CSF, and their combination on LLC tumor angiogenesis. The PECAM-1-positive vascular surface area was identified by immunohistochemistry. Only the combination, but not either sole therapy, could reduce the tumor angiogenesis. (*n* = 6/group). (**D**) Effects of anti-VEGF, capecitabine, and their combination on LLC tumor angiogenesis. The PECAM-1-positive vascular surface area was identified by immunohistochemistry. Note that only the combination, but not either sole therapy, could reduce the tumor angiogenesis, similarly to the findings to shown in panel (C). Data are mean ± SEM. N.S.: not significant, ^*^P < 0.01, and ^#^*P* < 0.05. (*n* = 5–6/group).

We screened for cytokines/chemokines expressed by LLC tumors *in vivo* that stimulate anti-VEGF-mediated PMN-MDSC recruitment. G-CSF and CCL2, but not IL-17A or Bv8, were increased by the anti-VEGF treatment, and capecitabine reduced those upregulations (Figure 3A). The selective antagonist of the CCL2 receptor CCR2 (RS102895) at the appropriate dose [18] did not affect either tumor growth or PMN-MDSC accumulation (data not shown). Anti-mouse G-CSF-neutralizing mAb inhibited tumor growth and PMN-MDSC accumulation (Figure 3B) under anti-VEGF administration, confirming G-CSF promotes intratumoral PMN-MDSC recruitment during anti-VEGF therapy [11]. G-CSF expression increased after anti-VEGF treatment in LLC tumor lysate, while IL-17A expression was low (Figure 4A). Bv8 protein was detected, and neither anti-VEGF nor capecitabine treatment changed its protein level (Figure 4B).

We focused on an Bv8-independent mechanism of tumor angiogenesis, as an IL-17-mediated immune-related mechanism appears unlikely. We assessed the platelet and endothelial cell adhesion molecule (PECAM)-1-positive vascular surface area in each tumor using an image analyzer. Anti-VEGF therapy alone did not inhibit tumor angiogenesis, whereas the addition of anti-G-CSF neutralizing antibody reduced the vascular surface area (Figure 4C). Tumor sections treated with anti-VEGF combined with capecitabine displayed similar results (Figure 4D), suggesting that an anti-VEGF/PMN-MDSCs/G-CSF axis may contribute to the Bv8-independent angiogenic escape induced by anti-VEGF treatment.

### Intratumoral MDSCs have PyNPase activities and the proangiogenic factor TP

**Figure 5:**
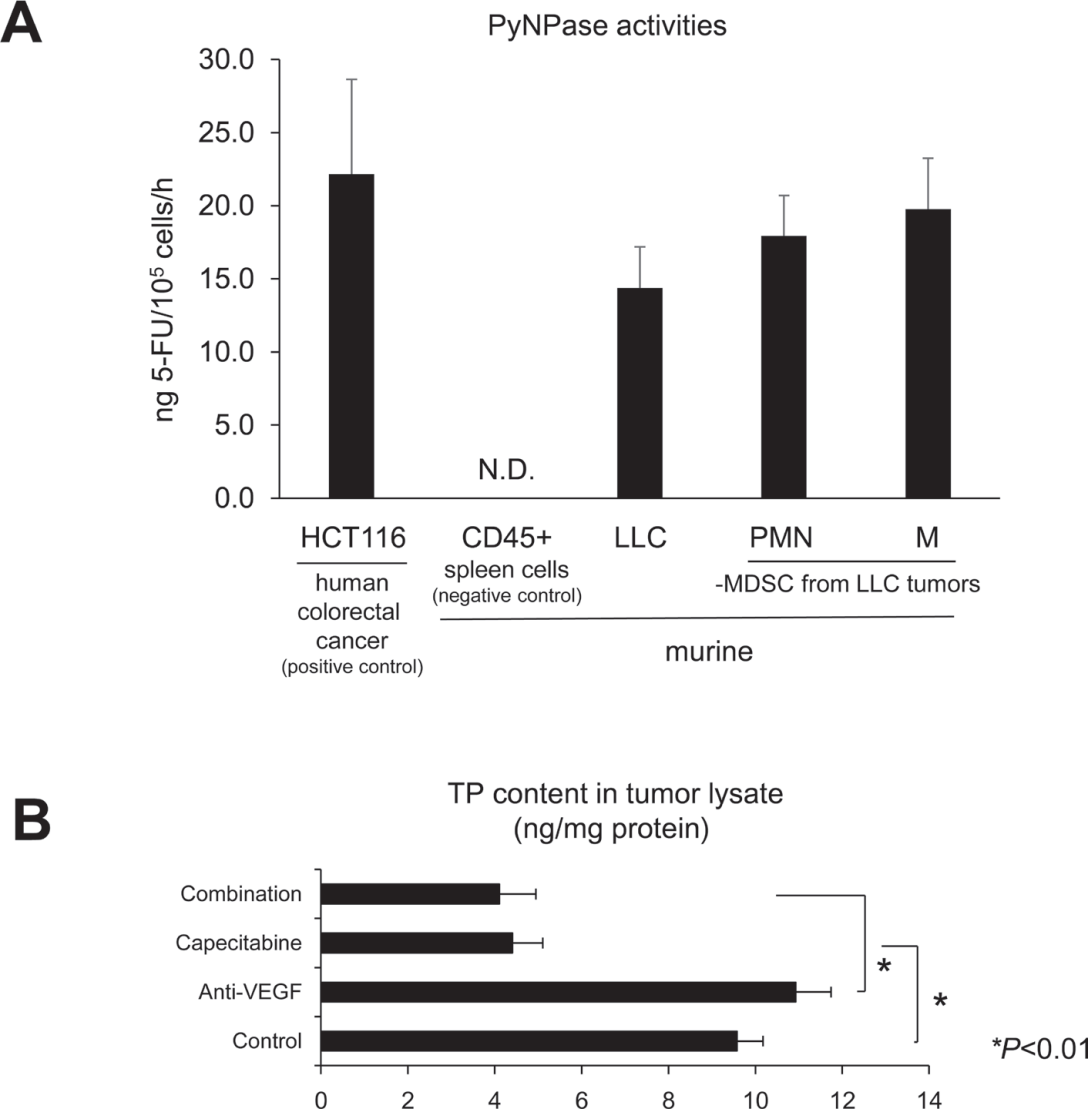
Intratumoral MDSCs have PyNPase activities and thymidine phosphorylase (TP), which is known as a proangiogenic factor (**A**) High PyNPase activity in LLC cells as well as both PMN-/M-MDSCs isolated from LLC tumors *in vivo*. PyNPase activity was assessed by converting efficiency to 5-FU. All data are shown as the mean ± SEM (*n* = 3–5/group). (**B**) Capecitabine reduced TP contents in LLC tumor lysate assessed by ELISA. All data are shown as the mean ± SEM. N.S.: not significant, ^*^P < 0.01. (*n* = 6/group).

Pyrimidine nucleotide phosphorylases (PyNPases), composed of thymidine phosphorylase (TP) and uridine phosphorylase (UP), are proangiogenic factors [19–21] and capecitabine converting enzymes [22, 23]. We investigated PyNPase activity in various cells, including MDSCs. The capecitabine-sensitive human colorectal cancer cell line HCT116 [22] demonstrated high PyNPase activity as a positive control, whereas CD45^+^ cells derived from murine spleen cells showed no activity (Figure 5A). The LLC cells and PMN- and M-MDSCs sorted from the LLC tumors had high PyNPase activity. The *in vivo* capecitabine treatment reduced the intratumoral content of TP independent of anti-VEGF treatment (Figure 5B), indicating that capecitabine could eliminate PyNPases in tumors.

### Impairment of capecitabine effects under anti-VEGF in PyNPases TP^−/−^/UP^−/−^ double-deficient mice

**Figure 6:**
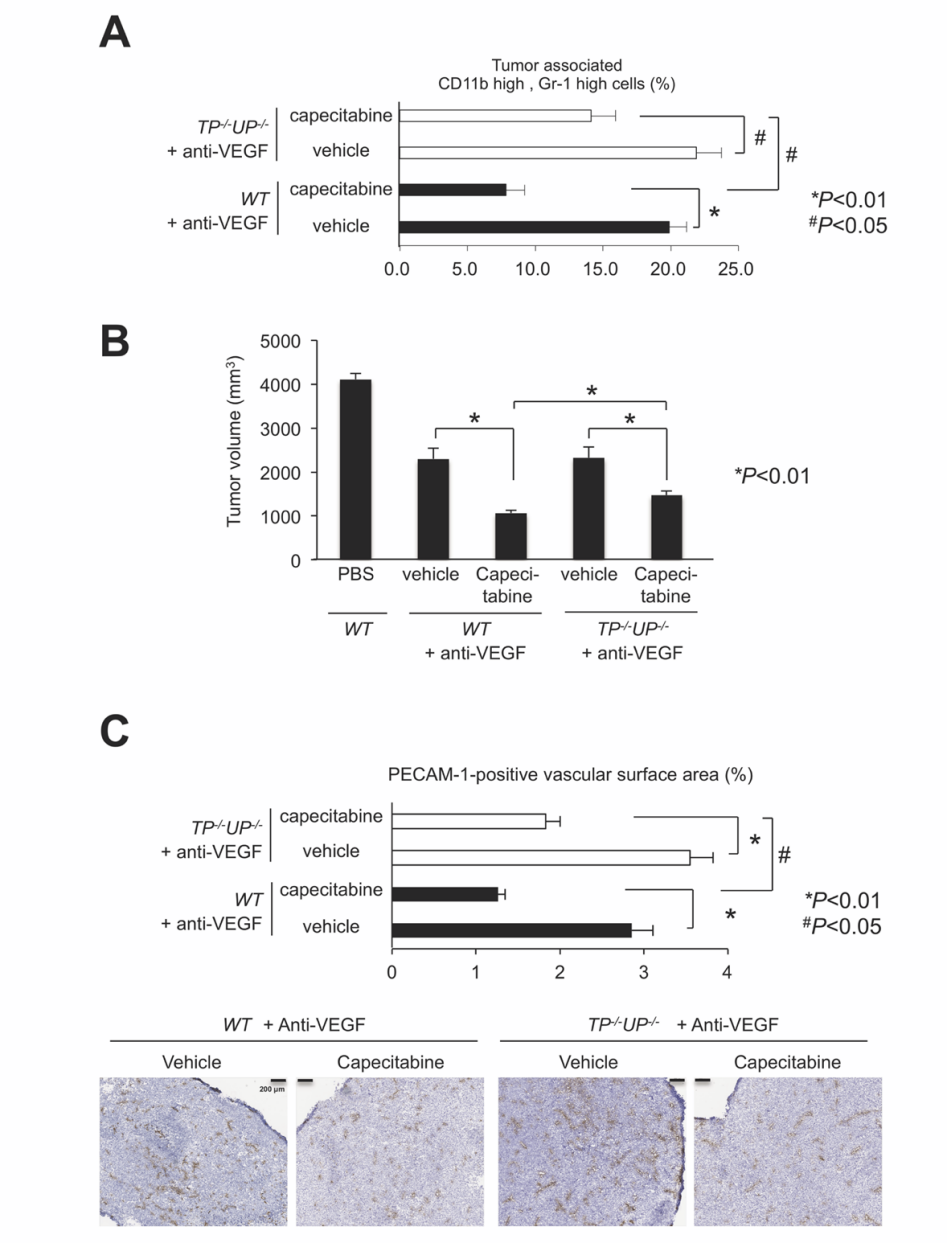
Impairment of capecitabine’s effects in *PyNPases TP*^*−/−*^/*UP*^−/−^ double deficient mice (**A**) Capecitabine-mediated reduction of PMN-MDSC infiltration during anti-VEGF therapy was impaired in PyNPases *TP*^*−/−*^*/UP*^*−/−*^ double deficient mice. All data are shown as the mean ± SEM. N.S.: not significant, ^*^P < 0.01 and ^#^*P* < 0.05. (*n* = 6/group). (**B**) Capecitabine-mediated reduction of tumor volume during anti-VEGF therapy was also impaired in PyNPases *TP*^*−/−*^*/UP*^*−/−*^ double deficient mice. All data are shown as the mean ± SEM. N.S.: not significant, ^*^P < 0.01 (*n* = 6/group). (**C**) Capecitabine-mediated reduction of tumor angiogenesis during anti-VEGF therapy was also impaired in PyNPases *TP*^*−/−*^*/UP*^*−/−*^ double deficient mice. All data are shown as the mean ± SEM. N.S.: not significant, ^*^P < 0.01 and ^#^*P* < 0.05. (*n* = 6/group).

We assessed TP activity in host-originated PMN-MDSCs, using LLC tumors bearing *TP*^*−/−*^/*UP*^*−/−*^ double-deficient mice [24] under anti-VEGF treatment. The capecitabine-dependent reduction of intratumoral PMN-MDSC recruitment in LLC tumors of the wild-type mice was suppressed in the LLC tumors of the *TP*^*−/−*^/*UP*^*−/−*^ double-deficient mice (Figure 6A). Capecitabine’s enhanced antitumor effect and restoration of antitumor angiogenesis under anti-VEGF therapy observed in the wild-type mice was reduced in the *TP*^*−/−*^/*UP*^*−/−*^ mice (Figure 6B and 6C, respectively).

## DISCUSSION

Our study produced three key observations. (*i*) Anti-VEGF therapy accelerated intratumoral CD11b^high^/Gr-1^high^ PMN-MDSC accumulation in anti-VEGF-resistant LLC tumors, but capecitabine diminished this effect and restored the antitumor activity of anti-VEGF treatment. (*ii*) The beneficial effect of capecitabine on LLC with anti-VEGF therapy is likely promoted by G-CSF expression, not IL-17 or Bv8. (*iii*) PyNPases, expressed by PMN-MDSCs, stimulated tumor resistance to anti-VEGF treatment.

The potential role of TP (platelet-derived endothelial cell growth factor [PD-ECGF]) in tumor angiogenesis in VEGF-negative tumor tissue was first suggested for colon cancer [19, 25, 26]. Since then, a number of studies have reported various tumor types and tumor infiltrating macrophages (TAMs) and lymphocytes to be the cellular sources of TP [27]. The present study is the first to determine that MDSCs, in particular the PMN type, are the functionally essential TP-expressing cells in tumors during anti-VEGF therapy.

Considering our present findings and the good safety and efficacy profiles of the combination of capecitabine and bevacizumab in phase III trials [4, 28], the observed partial effect of capecitabine on the restoration of anti-angiogenesis and inhibition of tumor growth during anti-VEGF treatment suggests more study may enable further optimization of anti-VEGF therapy to provide greater clinical benefit.

The capecitabine dose used in this study almost eliminated PMN-MDSCs in peripheral blood, but this effect was partially reduced in intratumoral areas (Figure 2). This may be due to (i) an insufficient distribution of capecitabine to the whole tumor tissue even though the systemic dose of it was sufficient, and/or (ii) the recruitment of other cells that are insensitive to capecitabine/5-FU and express TP/UP to tumor microenvironment. The latter might be likely, because CD68-positive tumor-associated macrophages (TAMs) were identified as the dominant cell source of TP/PD-ECGF in human colorectal cancer tissue [25]. Capecitabine reversed tumor escape from anti-VEGF, and may be a favorable chemotherapeutic agent that should be combined with bevacizumab in clinical settings (Supplementary Figure 1).

## MATERIALS AND METHODS

### Cell lines and culture conditions

The murine lung cancer (LLC) and murine melanoma (B16F1) cell lines were obtained in 2004 from the American Type Culture Collection (ATCC; Rockville, MD). The human colorectal cancer cell line HCT116 was obtained in 1990 from the ATCC. The LLC and B16F1 cells were cultured in high-glucose Dulbecco’s Modified Medium (DMEM) supplemented with 10% fetal bovine serum (FBS) and 4 mM glutamine, and maintained at 37° C in 5% CO_2_. The HCT116 was cultured in McCoy’s 5A Medium supplemented with 10% FBS and maintained at 37° C in a 5% CO_2_. All cell lines were passaged up to 20 times. All cell lines were authenticated using STR analyses, and were routinely tested for mycoplasma contamination using PCR-based detection methods in Central Institute for Experimental Animals (Kanagawa, Japan).

### Mice

Male 5- to 9-week-old C57BL/6 mice were obtained from KBT Oriental (Charles River Grade, Tosu, Saga, Japan). Male 7-week-old thymidine phosphorylase and uridine phosphorylase double-deficient mice (*TP*^−/−^/*UP*^−/−^) on the C57BL/6 background were provided by Prof. Furukawa (Department of Cancer Chemotherapy, Kagoshima University, Kagoshima) [24]. The mice were kept under specific pathogen-free and humane conditions, and the animal experiments were reviewed and approved by the Institutional Animal Care and Use Committee and by the Biosafety Committee for Recombinant DNA Experiments of Kyushu University (approval ID: A26–240–0). These experiments were also done in accordance with the recommendations for the proper care and use of laboratory animals and according to The Law (No. 105) and Notification (No. 6) of the Japanese Government, and the U.S. National Institutes of Health (NIH) Guide for the Care and Use of Laboratory Animals.

### Tumor models

LLC and B16-F1 tumor cells (5.0 × 10^6^) in 200 μL of DMEM were subcutaneously injected in the left flank of C57BL/6 mice or *TP*^−/−^/*UP*^−/−^ mice. The administration of anticancer agents was started when the tumor volumes reached 50 to 300 mm^3^. Anti-mouse VEGF-A monoclonal antibody (mAb) B20-4.1.1 (Genentech, Oceanside, CA) was intraperitoneally administered to the mice at the dose of 5 mg/kg weekly. Anti-mouse G-CSF mAb (clone 67604, rat IgG1, R&D Systems, Minneapolis, MN) was administered intraperitoneally at the dose of 10 µg/head daily. Capecitabine (pentylN-[1-[(2R,3R,4S,5R)-3,4-dihydroxy-5-methyloxolan-2-yl]-5-fluoro-2- oxopyrimidin-4-yl]carbamate, Chugai Pharmaceutical, Tokyo) and CCR2 antagonist (1′-[2-[4-(trifluoromethyl)phenyl]ethyl]spiro[1H-3,1-benzoxazine-4,4′-piperidine]-2-one, RS102895, Sigma-Aldrich, St. Louis, MO) were orally administered daily at the dose of 718 mg/kg and 10 mg/kg, respectively. 5-Fluorouracil (5-fluoro-1H-pyrimidine- 2,4-dione, 5-FU, Invitrogen, Carlsbad, CA) was administered intraperitoneally at the dose of 50 mg/kg twice a week. All animal experiments were conducted in accord with the institutional Animal Care and Use Committee. Tumor volume was estimated from the equation (L × W^2^ × 0.5); L = length and W = width.

### Flow cytometric analysis

Tumors were excised from control- and anti-VEGF-treated mice, and single cell suspensions were obtained by mincing tumors and homogenizing by disruption and digestion with a gentle MACS™ Dissociator and Tumor Dissociation Kit for mouse (Miltenyi Biotec, Bergisch Gladbach, Germany). Peripheral blood was pretreated with VersaLyse™ lysing solution for red blood cells lysis (Beckman Coulter, Indianapolis, IN).

Cells were stained with the following FITC-, PE-, PE-Cy5-, BV785, or Alexa647-conjugated monoclonal antibodies: mouse CD11b, Gr1, CD45 (BioLegend, San Diego, CA), G-CSF (eBioscience, San Diego, CA), and TP (Proteintech, Chicago, IL). The appropriate conjugated isotype-matched Immunoglobulin Gs (IgGs) were used for control. Intracellular cytokine staining was performed with the Cell Fixation/Permeabilization kit (BD Biosciences, Franklin Lakes, NJ). Cells were analyzed using a FACSAria™ cell sorter and an LSRFortessa™ cell analyzer (BD Biosciences) and FlowJo 7.6 software (Tree Star, San Carlos, CA).

### Immunohistochemistry

We evaluated microvessel density in the tumor tissue by performing immunohistochemical staining of PECAM-1 (rat anti-mouse PECAM-1 mAb, clone MEC 13.3; BD Biosciences). Tumor samples were collected at the end of the study. Immunohistochemistry was performed as described previously [29]. The microvessel density (%) was calculated from the ratio of the PECAM-1-positive staining area to the total observation area in the viable region. Positive staining areas were calculated using the imaging analysis software Tissue Studio^®^ (Definiens, Munich, Germany).

### Immunoassays

Tumor homogenates collected at the end of the study were analyzed with the BD™ Cytometric Bead Array (BD Biosciences). The concentrations of mouse IL-17A, G-CSF, CXCL12, CXCL5, and CCL2 were measured by a Quantikine ELISA kit (R&D Systems). Bv8 was quantified using the mouse prokineticin-2 (Bv8) ELISA kit (Cusabio Biotech, Selangor, Malaysia). The protein amount of TP was quantified using a specific ELISA kit (Cloud-Clone, Katy, TX).

### PyNPase enzymatic activity assay

Cells were homogenized in 10 mM Tris buffer (pH 7.4), containing 15 mM NaCl, 1.5 mM MgCl_2_, and 50 μM potassium phosphate. The homogenate was then centrifuged at 105,000 *g* for 90 min. The supernatant was dialyzed overnight against 20 mM potassium phosphate buffer (pH 7.4) containing 1 mM β-mercaptoethanol and used as a source of crude enzyme. All procedures were carried out below 4° C. The reaction mixture (120 μl) for the assay of the enzyme activity contained 183 mM potassium phosphate (pH 7.4), 10 mM 5′-dFUrd, and the crude enzyme from cells. The reaction was carried out at 37° C for 60 min, and then terminated by adding 360 μl of methanol. After removal of the precipitate by centrifugation, the amount of 5-FUra produced in the supernatant was measured with the My5-FU assay (Saladax Biomedical, Bethlehem, PA). The dThdPase and UPase activities are expressed as ng of 5-FUra converted/10^5^ cells/h.

### Statistical analysis

All data are expressed as the mean ± standard error of the mean (SEM). The Wilcoxon test was used, and *P*-values < 0.05 were accepted as significant. Statistical analyses were carried out using JMP, version 10 (SAS Institute, Cary, NC).

## SUPPLEMENTARY MATERIALS FIGURE



## Abbreviations

Bev: bevacizumab
FDA: U.S. Food and Drug Administration
VEGF: vascular endothelial growth factor
VEGFR: vascular endothelial growth factor receptor
MDSCs: myeloid-derived suppressor cells
PyNPase: pyrimidine nucleotide phosphorylase
TP: thymidine phosphorylase
UP: uridine phosphorylase
PMN: polymorphonuclear
LLC: Lewis lung carcinoma
5-FU: 5- fluorouracil
G-CSF: granulocyte-colony stimulating factor
IL-17: interleukin-17
PFS: progression-free survival
OS: overall survival
CCL2: chemokine (C-C motif) ligand 2
CCR2: C-C chemokine receptor type 2
PECAM-1: platelet and endothelial cell adhesion molecule-1
PD-ECGF: platelet-derived endothelial cell growth factor; TAMs: tumor infiltrating macrophages
