# Supplementary material for: Capecitabine reverses tumor escape from anti-VEGF through the eliminating CD11bhigh/Gr1high myeloid cells
Source: Oncotarget. 2018 Apr 3;9(25):17620–30. doi: 10.18632/oncotarget.24811 (PMC5915143; doi:10.18632/oncotarget.24811)
Supplement: Supplementary file 1 [file oncotarget-09-17620-s001.pdf]

Capecitabine reverses tumor escape from anti-VEGF through the eliminating CD11b^{high}/Gr1^{high} myeloid cells

SUPPLEMENTARY MATERIALS

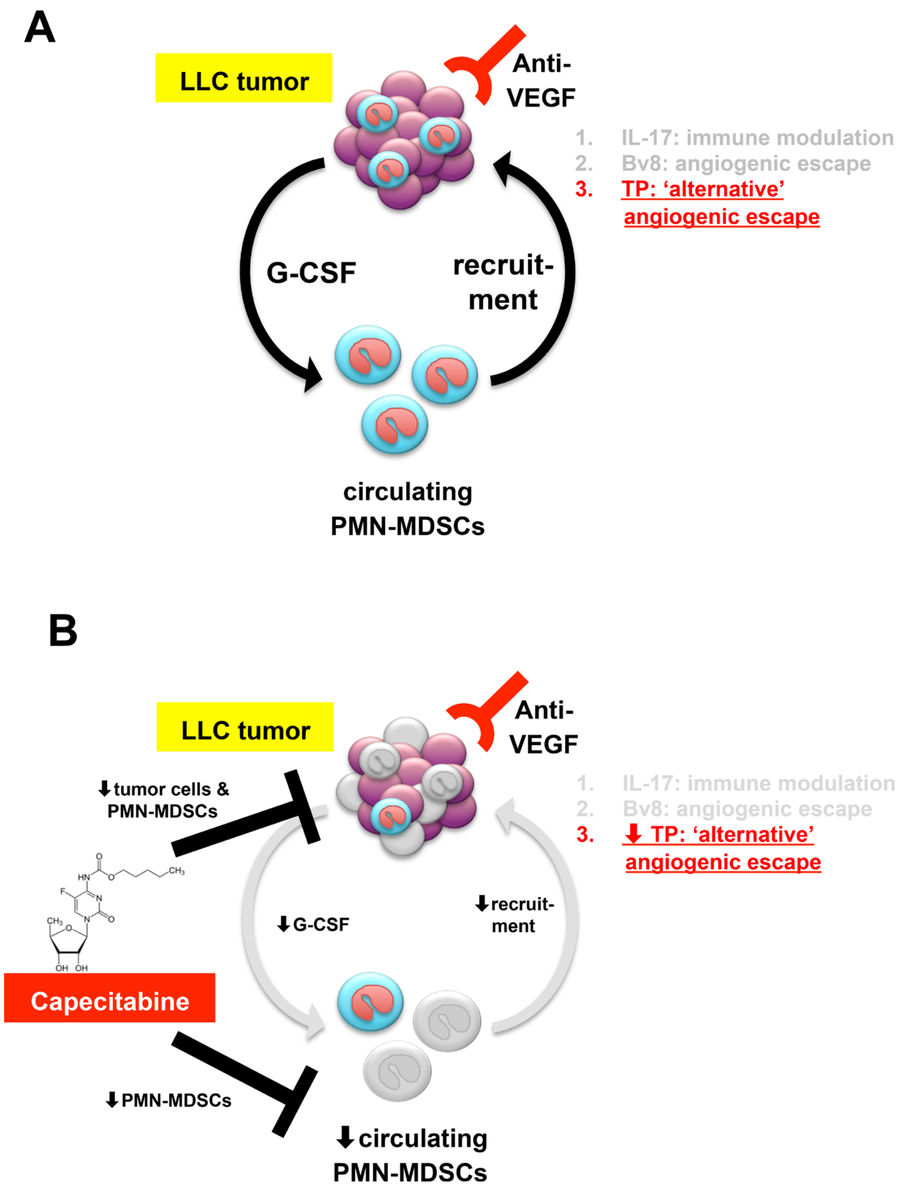

Supplementary Figure 1: Hypothetical scheme for overcoming resistance to anti-VEGF by capecitabine. (**A**) TP expressed by PMN-MDSCs as a new molecular target as a major cause of the bypass function for a tumor proangiogenic pathway under anti-VEGF therapy. (**B**) capecitabine eliminates TP-expressing PMN-MDSCs from both tumors and peripheral blood.
